# Mucinous Prostate Cancer Shows Similar Prognosis to Typical Prostate Acinar Carcinoma: A Large Population-Based and Propensity Score-Matched Study

**DOI:** 10.3389/fonc.2019.01467

**Published:** 2020-01-09

**Authors:** Feng Zhao, Xiaokai Yu, Mengyou Xu, Sunyi Ye, Shoumei Zang, Weixiang Zhong, Guoping Ren, Xin Chen, Senxiang Yan

**Affiliations:** ^1^Department of Radiation Oncology, The First Affiliated Hospital, College of Medicine, Zhejiang University, Hangzhou, China; ^2^Graduate School, College of Medicine, Zhejiang University, Hangzhou, China; ^3^Department of Urology, The First Affiliated Hospital, College of Medicine, Zhejiang University, Hangzhou, China; ^4^Department of Pathology, The First Affiliated Hospital, College of Medicine, Zhejiang University, Hangzhou, China; ^5^Institute of Pharmaceutical Biotechnology and The First Affiliated Hospital, College of Medicine, Zhejiang University, Hangzhou, China

**Keywords:** prostate cancer (PCa), mucinous PCa, incidence, radiation therapy (RT), surgery, prognosis

## Abstract

**Background:** Mucinous prostate cancer (PCa) is an extremely rare form of prostate malignancy. To date, the limited knowledge of its biology and outcomes stems from mostly small, single institution experiences. We analyzed the Surveillance, Epidemiology, and End Results (SEER) database to explore the incidence and treatment of mucinous PCa together with its prognostic factors to gain relatively large and consolidated insights.

**Methods:** Age-adjusted incidence (AAI) rates were evaluated over time. Propensity score matching (PSM) and Kaplan–Meier analyses were used to compare the prognosis between mucinous PCa and typical prostate acinar adenocarcinoma. We assessed cancer-specific survival (CSS) and overall survival (OS) after patient stratification according to summary stage and treatment choice. Cox hazards regression analysis was performed to determine independent predictors of CSS and OS.

**Results:** The AAI in 2016 was 0.24 per million. Patients with mucinous PCa had similar CSS and OS to matched individuals with typical prostate acinar adenocarcinoma. In terms of treatment, 65.3% of mucinous PCa patients underwent surgery, and 23.9% received radiation therapy. Patients who underwent surgery had longer survival (CSS, *p* = 0.012; OS, *p* < 0.001), and patients who received radiation therapy had similar survival to those who did not receive radiation therapy (CSS, *p* = 0.794; OS, *p* = 0.097). A multivariate Cox analysis for CSS and OS showed that older age (CSS: HR: 4.982, *p* = 0.001; OS: HR: 4.258, *p* < 0.001) and distant stage (CSS: HR: 40.224, *p* < 0.001; OS: HR: 9.866, *p* < 0.001) were independent prognostic factors for mucinous PCa patients.

**Conclusions:** Mucinous PCa has an extremely low AAI. Analysis of its outcomes indicates that it is not a more malignant tumor as previously suspected. Mucinous PCa shows a similar prognosis to typical prostate acinar carcinoma. Surgery was associated with prolonged survival. An older age at diagnosis and distant stage was associated with poor survival.

## Introduction

Mucinous adenocarcinoma of the prostate (previously referred to as colloid carcinoma of the prostate) is a rare morphological variant of prostate cancer (PCa) that is defined by the presence of at least 25% of the tumor being composed of glands with extraluminal mucin (1, 2). Approximately 0.2–0.4% of prostatic adenocarcinomas are diagnosed as mucinous adenocarcinoma (3–5). The overall low prevalence of mucinous prostatic adenocarcinoma has hindered our understanding of its clinical risk factors and prognosis. The clinical progression of mucinous adenocarcinoma of the prostate relative to typical acinar adenocarcinoma of the prostate, which accounts for more than 90% of PCa (6), remains controversial due to the scarce data from case reports or small case series (1, 4). Some studies have suggested that these tumors have a more aggressive behavior than typical acinar adenocarcinoma (7, 8); others have shown that these two tumor types to have similar outcomes (9, 10).

Because of conflicting survival data and the lack of population level data, we sought to determine the following: (1) a more accurate age-estimated incidence of mucinous PCa; (2) the general clinical characteristics of mucinous PCa; (3) whether mucinous PCa shows a more adverse prognosis than typical prostate acinar carcinoma; and (4) prognostic factors for cancer-specific survival (CSS) and overall survival (OS) in mucinous PCa; using the Surveillance, Epidemiology, and End Results (SEER) Program of the National Cancer Institute.

## Patients and methods

### Study Population

The current study relied on the SEER database, which covers ~26% of the United States (US) population. It is considered representative of the US in terms of demographic composition, cancer incidence and mortality. Two cohorts of patients were created using the SEER program (www.seer.cancer.gov) SEER^*^Stat Database. One cohort to estimate the incidence was created using the SEER 18 Registries Research Data + Hurricane Katrina Impacted Louisiana Cases, November 2018 Submission (2000–2016) <Katrina/Rita Population Adjustment>. The other cohort to estimate the patient demographics and survival was created using SEER 18 Registries Custom Data (with additional treatment fields), November 2018 Submission (1973–2016 varying).

The International Classification of Disease for Oncology, 3rd edition (ICD-O-3) was used to identify patients with mucinous PCa (ICD-O-3 codes: 8480/3 and 8481/3) and typical acinar adenocarcinoma of the prostate (ICD-O-3 code: 8140/3), which accounts for more than 90% of PCa (6). Mucinous PCa and typical prostate acinar carcinoma diagnosed between 2004 and 2016 by histologic confirmation either from biopsy or surgical pathology, rather than by clinical presentation, radiography, autopsy, or death records alone, were selected. In addition, we only included the patients with these tumor sequence numbers labeled “one primary only.”

### Variable Definitions

Covariates of interest extracted for each case included patients' demographic variables (age at diagnosis, race, and marital status), tumor grade (well-differentiated, moderately differentiated, poorly differentiated, undifferentiated, and unknown), SEER summary stage (localized, regional, and distant), prostate specific antigen (PSA), Gleason score, and treatment modality (surgery, radiation therapy, and chemotherapy).

### Statistical Analyses

Incidence rates were analyzed with SEER^*^Stat Software version 8.3.5 (Surveillance Research Program, National Cancer Institute, seer.cancer.gov/seerstat). Continuous data were compared using Student's *t*-test, and categorical data were compared using the Chi-square test. To adjust for differences between mucinous PCa and typical acinar adenocarcinoma of the prostate when comparing their prognoses, we performed a propensity score matching (PSM) analysis. The PSM model was based upon age, race, PSA, Gleason score, and SEER summary stage. Survival probabilities were estimated using the Kaplan–Meier method, and the log-rank test was used to assess any significant differences in CSS and OS stratified by each covariate. Cox proportional hazards models were used to analyze associations of patient characteristics and treatment modalities with patient survival. Only variables that were significantly associated with survival in the univariate Cox analysis were included in the multivariate Cox analysis. Hazard ratios (HRs) and 95% confidence intervals (CIs) were estimated using univariate and multivariate models. Cancer-specific mortality was further analyzed with competitive risk model, which was better than the Kaplan–Meier method and Cox regression model to some extent. Competitive risk model was performed with the “cmprsk,” “survival,” and “foreign” packages in R software (version 3.6.1). Statistical analysis was performed with SPSS Statistical Package version 25.0 (SPSS Inc., Chicago, IL, USA), and *p* < 0.05 was considered statistically significant.

## Results

### Incidence

**Figure 1:**
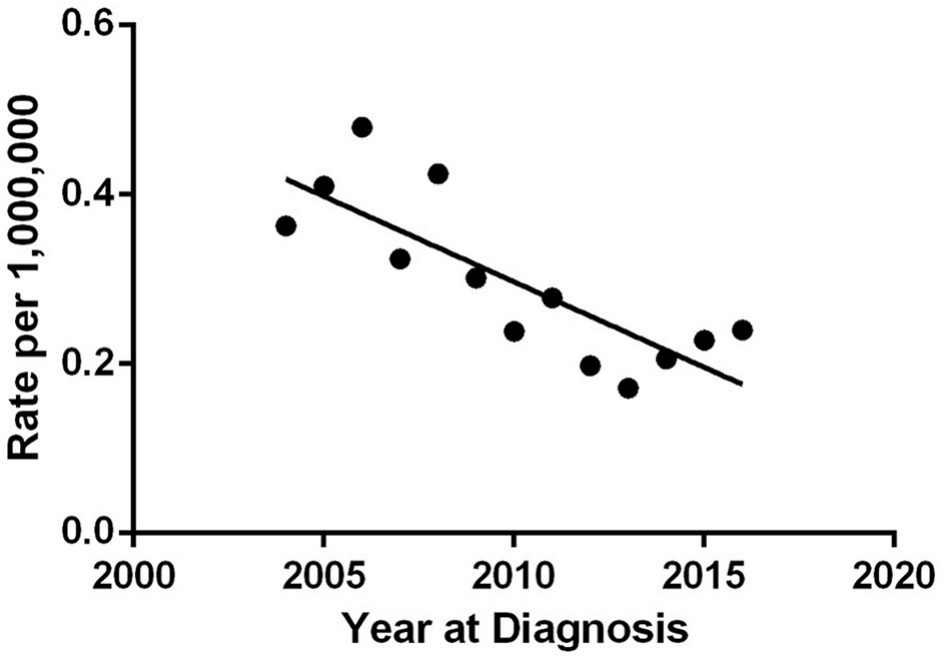
Age-adjusted incidence rate for mucinous PCa by year of diagnosis ranged from 0.36/1,000,000 in 2004 to 0.24/1,000,000 in 2016.

As shown in Figure 1, the age-adjusted incidence (AAI) of mucinous PCa over time decreased from 0.3621 per 1,000,000 in 2004 to 0.2393 per 1,000,000 in 2016, with a declining trend. Additionally, as shown in Supplementary Table 1, the percentage of cases of mucinous PCa relative to those of total PCa was between 0.04 and 0.09% over time.

### Patient Characteristics

A total of 360 patients diagnosed with mucinous PCa between 2004 and 2016 were identified from the SEER database. Demographic data for mucinous PCa patients is shown in Table 1. The mean age at diagnosis was 62.77 ± 9.84 years. The majority of patients (79.4%) were white. Most subjects (64.7%) were married. The majority of patients (70.3%) had poorly differentiated tumors. Among 360 included cases, 251 (69.7%), 90 (25.0%), and 18 (5.0%) cases were categorized as localized, regional, and distant stages, respectively. The serum PSA levels of mucinous PCa patients were more frequently <10 ng/ml (55.8%). Nearly half of the patients (49.2%) lacked Gleason score records, and among the remaining 183 patients with Gleason scores information, patients more frequently had a Gleason score of 3 + 4 = 7 or 4 + 3 = 7 (57.9%). Regarding treatment, 65.3% of patients were managed with surgery, 23.9% received radiation therapy, and 6.9% received radiation therapy after surgery.

**Table 1:** Patient demographics and clinical characteristics (*n* = 360).

**Characteristics**	**Level**	**Number (%)**
Age at diagnosis	Mean ± SD	62.77 ± 9.84
	Median (range)	62 (40–90)
Race	White	286 (79.4%)
	Black	51 (14.2%)
	Others/unknown	23 (6.4%)
Marital status	Married	233 (64.7%)
	Unmarried	88 (24.4%)
	Unknown	39 (10.8%)
Tumor grade	Well-differentiated	6 (1.7%)
	Moderately differentiated	80 (22.2%)
	Poorly differentiated	253 (70.3%)
	Undifferentiated	2 (0.6%)
	Unknown	19 (5.3%)
Summary stage	Localized	251 (69.7%)
	Regional	90 (25.0%)
	Distant	18 (5.0%)
	Unknown	1 (0.3%)
PSA (ng/ml)	<10	201 (55.8%)
	≥10~ <20	62 (17.2%)
	≥20	57 (15.8%)
	Unknown	40 (11.1%)
Gleason score	≤6	32 (8.9%)
	7	106 (29.4%)
	≥8 or primary = 5	45 (12.5%)
	Unknown	177 (49.2%)
Surgery	Yes	235 (65.3%)
	None/unknown	125 (34.7%)
Radiation therapy	Yes	86 (23.9%)
	RT after surgery	25 (6.9%)
	None/unknown	274 (76.1%)
Chemotherapy	Yes	5 (1.4%)
	No/unknown	355 (98.6%)

*PSA, prostate specific antigen; RT, Radiation therapy*.

The demographic and clinical characteristics were compared across summary stages (Table 2) and surgical treatment (Table 3). According to stratification by summary stage, there were no significant differences among mucinous PCa patients in terms of age, marital status, Gleason score, or radiation therapy. However, patients who presented with distant disease were more likely to be black (*p* = 0.002), more likely to have a serum PSA level ≥20 ng/ml (*p* < 0.001), less likely to have low tumor grade (well-differentiated or moderately differentiated tumors) (*p* < 0.001), and less likely to undergo surgical resection (*p* < 0.001). According to stratification by surgical treatment, there were no significant differences among mucinous PCa patients in terms of Gleason score (*p* = 0.085). However, patients who underwent surgical resection were more likely to be younger (*p* < 0.001), white (*p* = 0.01), and married (*p* < 0.001), more likely to have serum PSA levels <10 ng/ml (*p* < 0.001), and less likely to receive radiation therapy (*p* < 0.001).

**Table 2:** Patient characteristics by SEER summary stage (*n* = 359).

		**Summary stage**			
**Characteristics**	**Level**	**Localized (*n* = 251)**	**Regional (*n* = 90)**	**Distant (*n* = 18)**	***P-*value**
Age at diagnosis	Mean ± SD	62.60 ± 10.39	62.34 ± 7.89	67.17 ± 10.38	
	Median (range)	62 (40–90)	62 (43–82)	64.5 (51–85)	
	≤65	160 (63.7%)	59 (65.6%)	10 (55.6%)	0.723
	>65	91 (36.3%)	31 (34.4%)	8 (44.4%)	
Race	White	202 (80.5%)	76 (84.4%)	7 (38.9%)	0.002
	Black	34 (13.5%)	8 (8.9%)	9 (50.0%)	
	Others/unknown	15 (6.0%)	6 (6.7%)	2 (11.1%)	
Marital status	Married	157 (62.5%)	64 (71.1%)	11 (61.1%)	0.014
	Unmarried	59 (23.5%)	22 (24.4%)	7 (38.9%)	
	Unknown	35 (13.9%)	4 (4.4%)	0 (0.0%)	
Tumor grade	Low	70 (27.9%)	16 (17.8%)	0 (0.0%)	<0.001
	High	172 (68.5%)	71 (78.9%)	11 (61.1%)	
	Unknown	9 (3.6%)	3 (3.3%)	7 (38.9%)	
PSA (ng/ml)	<10	144 (57.4%)	52 (57.8%)	5 (27.8%)	<0.001
	≥10~ <20	53 (21.1%)	8 (8.9%)	1 (5.6%)	
	≥20	25 (10.0%)	20 (22.2%)	12 (66.7%)	
	Unknown	29 (11.6%)	10 (11.1%)	0 (0.0%)	
Gleason score	≤6	26 (10.4%)	3 (3.3%)	3 (16.7%)	0.021
	3 + 4/4 + 3	81 (32.3%)	24 (26.7%)	1 (5.6%)	
	≥8 or primary = 5	28 (11.2%)	13 (14.4%)	4 (22.2%)	
	Unknown	116 (46.2%)	50 (55.6%)	10 (55.6%)	
Surgery	Yes	153 (61.0%)	79 (87.8%)	3 (16.7%)	<0.001
	None/unknown	98 (39.0%)	11 (12.2%)	15 (83.3%)	
Radiation therapy	Yes	53 (21.1%)	25 (27.8%)	7 (38.9%)	0.150
	None/unknown	198 (78.9%)	65 (72.2%)	11 (61.1%)	
Chemotherapy	Yes	1 (0.4%)	2 (2.2%)	2 (11.1%)	0.019
	No/unknown	250 (99.6%)	88 (97.8%)	16 (88.9%)	

*One patient with unknown stage was excluded in this analysis. Low tumor grade, Well and moderately differentiated tumor grade; High, Poorly differentiated and undifferentiated tumor grade; PSA, prostate specific antigen; The p-value was calculated by the chi-square test for categorical covariates*.

**Table 3:** Patient characteristics by surgery treatment (*n* = 360).

**Characteristics**	**Level**	**Surgery (*n* = 235)**	**Non-surgery/unknown (*n* = 125)**	***P*-value**
Age	Mean ± SD	59.94 ± 8.69	68.09 ± 9.69	<0.001
	Median (range)	60 (40–88)	67 (48–90)	
	≤65	171 (72.8%)	59 (47.2%)	<0.001
	>65	64 (27.2%)	66 (52.8%)	
Race	White	197 (83.8%)	89 (71.2%)	0.010
	Black	24 (10.2%)	27 (21.6%)	
	Others/unknown	14 (6.0%)	9 (7.2%)	
Marital status	Married	175 (74.5%)	58 (46.4%)	<0.001
	Unmarried	49 (20.9%)	39 (31.2%)	
	Unknown	11 (4.7%)	28 (22.4%)	
Tumor grade	Low	58 (24.7%)	28 (22.4%)	0.028
	High	170 (72.3%)	85 (68.0%)	
	Unknown	7 (3.0%)	12 (9.6%)	
PSA (ng/ml)	<10	148 (63.0%)	53 (42.4%)	<0.001
	≥10~ <20	43 (18.3%)	19 (15.2%)	
	≥20	24 (10.2%)	33 (26.4%)	
	Unknown	20 (8.5%)	20 (16.0%)	
Gleason score	≤6	17 (7.2%)	15 (12.0%)	0.085
	7	75 (31.9%)	31 (24.8%)	
	≥8 or primary = 5	24 (10.2%)	21 (16.8%)	
	Unknown	119 (50.6%)	58 (46.4%)	
Radiation therapy	Yes	22 (9.4%)	64 (51.2%)	<0.001
	None/unknown	213 (90.6%)	61 (48.8%)	

*Low tumor grade, Well and moderately differentiated tumor grade; High tumor grade, Poorly and undifferentiated tumor grade; PSA, prostate specific antigen; The p-value was calculated by the chi-square test for categorical covariates*.

### Patient Survival

**Figure 2:**
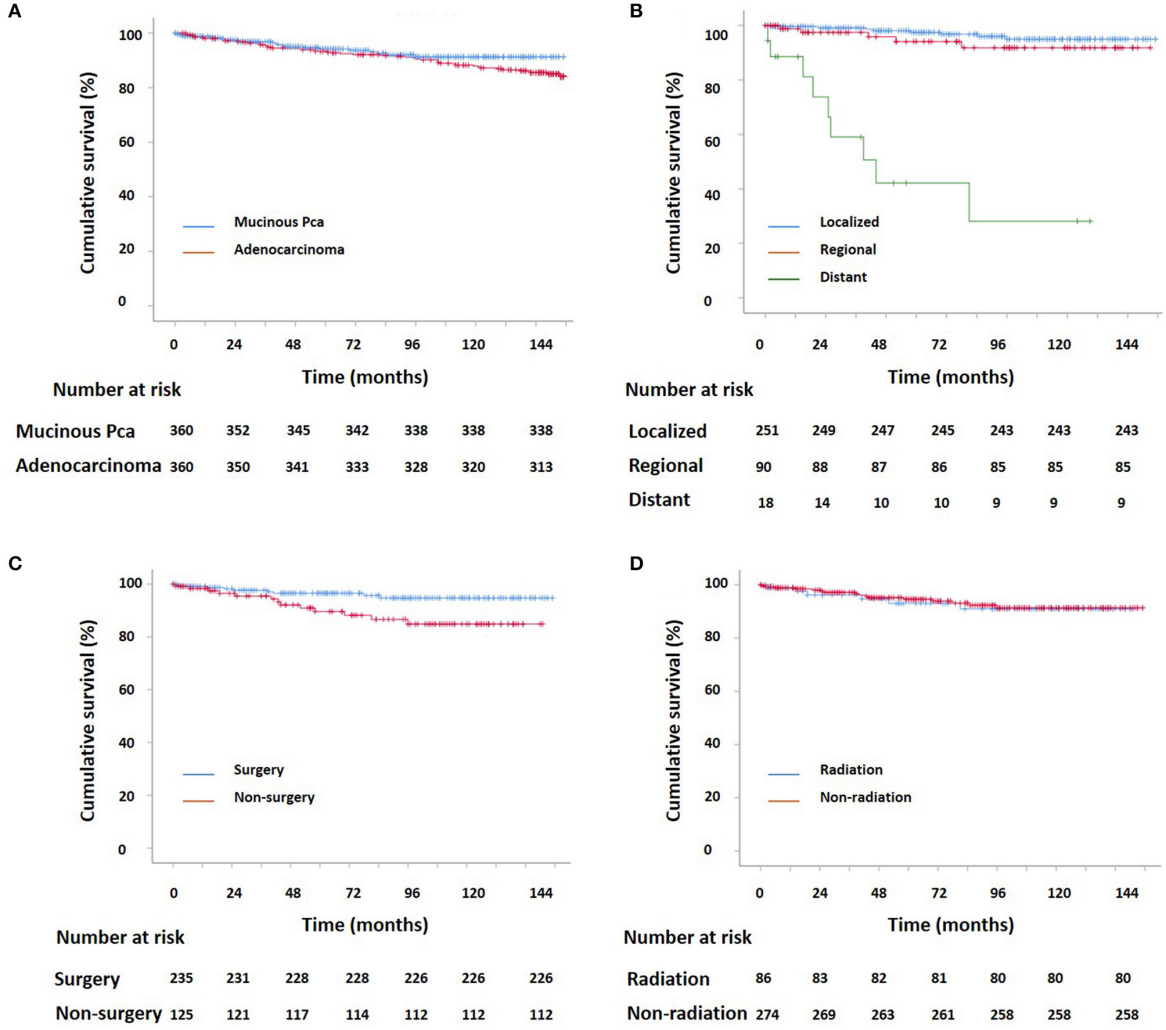
**(A)** Kaplan–Meier estimated CSS for patients with prostate adenocarcinoma and mucinous PCa (*p* = 0.23); **(B)** Kaplan–Meier estimated CSS for localized, regional, and distant mucinous PCa (localized vs. regional: *p* = 0.24; localized vs. distant: *p* < 0.001; regional vs. distant: *p* < 0.001); **(C)** Kaplan–Meier estimated CSS for mucinous PCa with and without surgery (*p* = 0.012); **(D)** Kaplan–Meier estimated CSS for mucinous PCa with and without radiation (*p* = 0.794).

**Figure 3:**
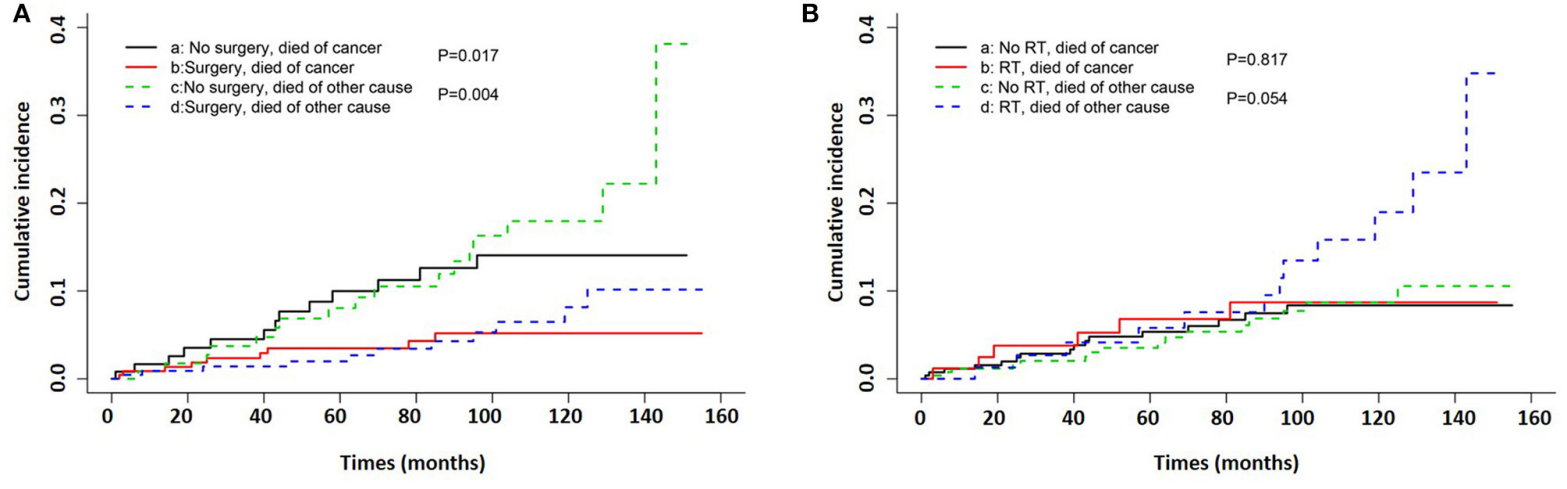
Competing risk curve of cancer-specific survival for patients with mucinous PCa. **(A)** competitive risk model of surgery, **(B)** competitive risk model of radiation therapy.

Kaplan–Meier curves for survival in patients with mucinous PCa and typical acinar adenocarcinoma of the prostate, stratified by SEER summary stage, surgery, and radiation therapy are shown in Figure 2 (CSS) and Supplementary Figure 1 (OS), and log-rank analysis revealed that patients with mucinous PCa had similar CSS and OS rates to matched patients with typical acinar adenocarcinoma of the prostate (CSS, *p* = 0.23; OS, *p* = 0.208). Patients with mucinous PCa who presented with distant disease had shorter survival than patients with localized and regional disease (CSS, *p* < 0.001; OS, *p* < 0.001); patients who underwent surgery had longer survival (CSS, *p* < 0.001; OS, *p* < 0.001); and patients who received radiation therapy had similar survival to patients who did not receive radiation therapy (CSS, *p* = 0.794; OS, *p* = 0.097). Additionally, on the competitive risk model of surgery (Figure 3A), patients who did not receive surgery were associated with significantly higher cancer-specific mortality than surgical patients (*p* = 0.017). While, patients who did not receive radiation therapy had similar cancer-specific mortality with radiation therapy patients (*p* = 0.817) (Figure 3B). Furthermore, Kaplan–Meier curves for CSS in patients with mucinous PCa, stratified by treatment of surgery & RT after surgery, only surgery, only RT, and no surgery & RT are shown in Supplementary Figure 2, and log-rank analysis revealed that only surgery and only RT had similar longer CSS than no surgery & RT (only surgery, *p* < 0.001; only RT, *p* = 0.045).

Variables potentially influencing CSS were analyzed using univariate and multivariate Cox proportional hazards analysis (Table 4), older age, PSA ≥20 ng/ml, distant stage, and no surgical treatment were associated with poor prognosis (all *p* < 0.05). In the multivariate analysis that included age, PSA, summary stage, and surgery as covariates, patients who were older (multivariate HR = 4.982, 95% CI: 1.920–12.930; *p* = 0.001) and had distant stage disease (multivariate HR = 40.224, 95% CI: 9.090–177.999; *p* < 0.001) exhibited worse survival outcomes. Interestingly, patients who did not undergo surgery exhibited similar survival outcomes to those who underwent surgery (multivariate HR 1.054, 95% CI: 0.358–3.108; *p* = 0.924), although they had significantly worse survival outcomes in the univariate analysis (HR = 2.819, 95% CI: 1.205–6.596; *p* = 0.017). Patients who had PSA levels ≥20 ng/ml exhibited similar survival outcomes to those with PSA levels <20 ng/ml (multivariate HR 1.144, 95% CI: 0.307–4.257; *p* = 0.841), although they had significantly worse survival outcomes in the univariate analysis (HR = 5.653, 95% CI: 2.103–15.199; *p* = 0.001). Similarly, variables potentially influencing OS were analyzed also using univariate and multivariate Cox proportional hazards analysis (Supplementary Table 2).

**Table 4:** Univariate and multivariate analyses for CSS of patients.

**Variables**	**Level**	**Univariate**		**Multivariate**	
		**HR (95% CI)**	***P-*value**	**HR (95% CI)**	***P*-value**
Age	≤65	1		1	
	>65	4.986 (1.950–12.749)	0.001	4.982 (1.920–12.930)	0.001
Race	White	1		/	/
	Black	1.885 (0.690–5.146)	0.216	/	/
	Others/unknown	0.750 (0.099–5.660)	0.781	/	/
Marital status	Married	1		/	/
	Unmarried	1.679 (0.660–4.271)	0.276	/	/
	Unknown	1.317 (0.371–4.669)	0.670	/	/
Tumor grade	Low	1		/	/
	High	2.915 (0.379–22.449)	0.304	/	/
	Unknown	48.995 (6.199–387.262)	<0.001	/	/
PSA (ng/ml)	<10	1		1	
	≥10~ <20	0.937 (0.194–4.510)	0.935	1.442 (0.286–7.274)	0.658
	≥20	5.653 (2.103–15.199)	0.001	1.144 (0.307–4.257)	0.841
	Unknown	2.938 (0.860–10.038)	0.086	3.298 (0.927–11.728)	0.065
Summary stage	Localized	1		1	
	Regional	1.943 (0.636–5.942)	0.244	2.288 (0.645–8.112)	0.200
	Distant	31.151 (11.826–82.052)	<0.001	40.224 (9.090–177.999)	<0.001
Gleason score	≤6	1		/	/
	3 + 4 & 4 + 3	0.290 (0.059–1.437)	0.130	/	/
	≥8	1.494 (0.373–5.974)	0.571	/	/
	Unknown	1.377 (0.360–5.262)	0.640	/	/
Surgery	Yes	1		1	
	None/unknown	2.819 (1.205–6.596)	0.017	1.054 (0.358–3.108)	0.924
RT	Yes	1		/	/
	None/unknown	0.882 (0.345–2.256)	0.794	/	/

*CSS, cancer-specific survival; HR, Hazard ratio; Low tumor grade: Well and moderately differentiated tumor grade; High tumor grade, Poorly and undifferentiated tumor grade; PSA, prostate specific antigen; RT, Radiation therapy*.

## Discussion

### Age-Adjusted Incidence Was Extremely Low

Owing to its rarity, very few reports have described this entity, and all contemporary articles are limited to sporadic cases or small sample case series. In the present study, we took advantage of the large data set from the SEER program to investigate the incidence of and prognostic factors for mucinous PCa in the largest series of cases of mucinous PCa reported to date. After adjusting for age, we calculated an incidence rate of <0.5 over million person-years for mucinous PCa over time, with a slightly declining trend. In addition, mucinous PCa accounted for <0.1% of all PCa, which is less than historical reports of 0.2–0.5% (3, 4). Due to its' rarity, therefore, the declining trend of age-adjusted incidence need to be further validated. Consequently, only a minority of practicing urologists will witness a single case of mucinous PCa in their clinical practice.

### Older Age at Diagnosis Was Associated With Poor Survival

According to our results, the mean age of patients with mucinous PCa at diagnosis was 62.77 years, which agrees with Samaratung et al.'s reports of an average age of 61.4 years (9). Our study demonstrated that older age (age > 65 years) was associated with worse prognosis, which is consistent with previous reports of typical PCa (11, 12). Our study may contribute to the prognosis prediction for this rare histological variant of PCa. The poor prognosis of older patients may be related to the fact that these patients tend to have poor Karnofsky Performance Status (KPS) scores; these patients with low KPS scores often cannot tolerate intensive treatment. Another reason for the worse prognosis is the propensity of higher grade and higher Gleason score in the older patients of mucinous PCa, from our preliminary experiment.

### Married Status Was Not Associated With Better Survival

Marital status has been increasingly recognized as an important factor in the survival of cancer patients (13, 14). In our study, the majority of patients who underwent surgical resection were married (74.5%), possibly due to married patients receiving social and financial support from their families, which tended to lead to the choice of a proactive treatment modality. As reported by previous studies, marital status is a prognostic factor for the survival of PCa patients, as being married was associated with better outcomes (14). However, we did not observe that marital status affected survival in mucinous PCa patients in our study. It might partly due to the selection bias from treating physicians that offer surgery to younger patients compared with offering RT to older patients (some older patients were classified as “unmarried” because of widowhood).

### Gleason Score and PSA Level Did Not Show More Aggressive Features as Previously Suspected

Whether a Gleason score should be assigned to mucinous PCa is a matter of debate, with some pathologists suggesting that all cases of mucinous PCa should be assigned a high Gleason score, while some pathologists support grading mucinous adenocarcinoma of the prostate on the basis of the underlying architectural pattern rather than assuming that all of these tumors are aggressive (1, 4). Additionally, the 2014 ISUP recommendations for grading mucinous cancers is to grade based upon the underlying architecture (15–17). In our study, the Gleason score was determined based on the underlying architecture. In fact, almost half of the patients lacked Gleason score information; for the remaining patients, the Gleason scores of the majority of patients were 3 + 4 = 7 or 4 + 3 = 7. In other words, our study revealed that mucinous PCa did not show more aggressive features as previously suspected. This phenomenon was consistent with the findings of a prior study reported by Samaratunga et al. (9).

Although mucinous PCa is associated with elevated PSA levels, most (57.4%) mucinous PCa patients presented with PSA levels <10 ng/ml, and a small number of patients showed PSA levels ≥20 ng/ml, consistent with prior reports (9, 10). For patients with distant stage disease, the mostly (66.7%) of patients showed PSA levels ≥20 ng/ml. Therefore, high serum PSA levels may be associated with the biological behavior of mucinous PCa, especially with metastatic status. The majority of mucinous PCa patients in our study presented with an early stage of localized (69.72%) resectable disease, consistent with prior reports (10). Our reported rate of distant metastasis for mucinous PCa (5.0%) was lower than that in prior case reports or case series (9, 18).

### Mucinous PCa Showed Similar Prognosis to Typical Prostate Adenocarcinoma

Previous studies remain controversial regarding the clinical progression of mucinous PCa. Several studies have considered this tumor to be an aggressive cancer that tends to develop bone metastases, with an associated poor outcome (6–8); several studies have considered that mucinous PCa is not more aggressive and may be even less aggressive than typical prostatic adenocarcinoma (9, 10). Therefore, to determine whether mucinous PCa shows a more adverse prognosis than typical prostate adenocarcinoma, we adjusted for differences in common prognostic predictors between mucinous PCa and typical prostate acinar carcinoma, including age, race, PSA, Gleason score, and SEER summary stage. Interestingly, when total 360 patients with mucinous PCa were compared with a control group of patients with typical prostate acinar adenocarcinoma, mucinous PCa patients had similar survival progression in terms of CSS and OS compared with those with typical prostate acinar carcinoma. Therefore, we can assume that the mucinous are clonally related to typical PCa. Similarly, Johnson et al. revealed that TMPRSS2-ERG is expressed in almost 50% of cases of mucinous PCa and PCa with mucinous features, similar to rates of expression in conventional PCa; therefore, this study strongly suggests that these rare subtypes of PCa are clonally related to conventional PCa (19).

Our research shows significantly better survival for patients diagnosed with a localized or regional disease than in those with distant disease. Multivariate Cox proportional hazards regression analysis also indicated that SEER summary stage with distant disease is an independent predictor of shorter CSS and OS. In other words, it is vital to make an early stage diagnosis for mucinous PCa. In addition, interestingly, in our cohort with distant disease, half of the patients were black (50.0%). While, due to the distribution of races in the Western population, white patients accounted for almost 80% of patients with non-distant disease (20). In other words, black patients with mucinous adenocarcinoma presented with more advanced stage disease than white patients, which was similar to the findings of a previous report (21).

### Surgery Was Associated With Better Survival

Regarding treatment for mucinous PCa, no clinical trial has been reported due to the rarity of this disease. Clinically, the treatment for mucinous PCa is similar to that for typical acinar adenocarcinoma, and includes androgen deprivation therapy (ADT), surgery, radiation therapy, chemotherapy, etc. In this study, surgery was the most commonly used treatment modality for non-metastatic mucinous PCa, which is consistent with the findings of a previous report (9). Kaplan–Meier curves analysis indicates that surgery increased CSS and OS. In addition, from the univariate Cox proportional hazards analysis, our study demonstrated that not receiving surgery was associated with poor prognosis in terms of CSS and OS. However, the multivariate Cox proportional hazards analysis revealed that patients who did not undergo surgery did not exhibit worse survival in terms of CSS and OS. This might be because the factors of older age and distant stage have more weight than a lack of surgery in the multivariate Cox proportional hazards analysis. Because it may exist a selection bias from treating physician that offer surgery to younger and more localized patients compared with radiation therapy.

Radiation therapy was only used in 21.1, 27.8, and 38.9% of cases for localized, regional, and distant stages of mucinous PCa. Younger (≤ 65 years) patients tended to choose surgery, and only ~10% of them received radiation therapy as adjuvant therapy. The percentage of patients treated with radiation therapy was relatively low and radiation therapy did not show significant benefits for prolonging CSS and OS. It might because the patients who received RT were more likely to older and distant stage; these two factors were associated with poor survival. Kaplan–Meier curves for CSS in patients with mucinous PCa stratified by treatment modality revealed that the patients who were treated with RT (RT after surgery and only RT) had similar CSS to the patients who treated with only surgery. Similarly, Guler et al. (22) reported that in mucinous PCa patients, treatment with radiation therapy and ADT contributed to a complete response. The association between RT and prognosis needs to be further verified. In our study, only five patients received chemotherapy, which was always used in the case of castration resistant PCa (CRPC). This finding might indicate that patients seldom develop CRPC over time. Generally, mucinous PCa responds well to hormonal therapy (22); however, ADT management information was not documented in the SEER database. Therefore, this study lacks information about ADT regarding the prognosis in terms of CCS and OS.

## Limitations

Similar to other studies that have utilized the SEER database as the data source, our study suffered certain limitations that require clarification for accurate interpretation of the results. Although the SEER data include information regarding the use of surgery, radiation therapy, and chemotherapy, the details of these therapies (i.e., surgical margins, radiation dose and chemotherapy regimen) are not recorded in the database. Additionally, hormone therapy, which may have an important role in the management of mucinous PCa, is not documented in the SEER database. In addition, almost half of the patients lacked information on Gleason scores, which are important for the evaluation of PCa. Finally, although this study examined one of the largest populations of men with mucinous PCa, the overall numbers are limited.

## Conclusions

This report presents the most comprehensive study of mucinous PCa rates and survival figures performed to data. Despite the rarity of mucinous PCa, we used a population-based approach and obtained the following insights: (1) Age-adjusted incidence of mucinous PCa was extremely low, with a slightly declining trend; (2) Mucinous PCa showed a similar prognosis to typical prostate acinar carcinoma; (3) As the most commonly used treatment strategy for non-metastatic mucinous PCa, surgery improved outcome of mucinous PCa, although it is not an independent prognosis of CCS and OS; (4) An older age at diagnosis and distant stage was associated with poor survival.

## Data Availability Statement

The datasets for this study are publicly available from SEER database. We get access to it via accession number: 10633-Nov 2018.

## Ethics Statement

The study was based on a secondary analysis of the previously collected, publicly available, and de-identified data. The SEER database holds no identifying patient information, all data are anonymous, therefore, the written informed consent is no need for this study. This investigation was conducted in accordance with the ethical standards, according to the Declaration of Helsinki, and according to national and international guidelines, and the institutional review board of our hospital approved this study.

## Author Contributions

FZ contributed to the literature search, study design, data collection, data analysis and interpretation, figures, and writing. XY, SYe, and MX contributed to data collection, data analysis and interpretation, and writing. SZ, WZ, and GR contributed to data collection, analysis, and interpretation. XC contributed to study design and data interpretation. SYa contributed to study design, data analysis, interpretation, figures, and writing. All authors read and approved the final version of the manuscript.

### Conflict of Interest

The authors declare that the research was conducted in the absence of any commercial or financial relationships that could be construed as a potential conflict of interest.

## References

[1] Osunkoya AO. Mucinous and secondary tumors of the prostate. Mod Pathol. (2018) 31:S80 10.1038/modpathol.2017.132 29297488

[2] Zhang L Zhang L Chen M Fang Q. Incidental discovery of mucinous adenocarcinoma of the prostate following transurethral resection of the prostate: a report of two cases and a literature review. Mol Clin Oncol. (2018) 9:432–6. 10.3892/mco.2018.1686 30214732 PMC6125698

[3] Moch H Humphrey PA Ulbright TM Reuter VE WHO Classification of Tumors of the Urinary System and Male Genital Organs. Lyon: IARC (2016). 10.1016/j.eururo.2016.02.028 26996659

[4] Enciu M Aşchie M Deacu M Poinăreanu I. Morphological characteristics of a mucinous adenocarcinoma of the prostate: differential diagnosis considerations. Rom J Morphol Embryol. (2013) 54:191. 23529329

[5] Prendeville S Nesbitt ME Evans AJ Fleshner NE van der Kwast TH. Variant histology and clinicopathological features of prostate cancer in men younger than 50 years treated with radical prostatectomy. J Urol. (2017) 198:79–85. 10.1016/j.juro.2017.01.061 28130102

[6] Mikuz G. Histologic classification of prostate cancer. Anal Quant Anal Quant Cytopathol Histpathol. (2015) 37:39–47. 26072633

[7] Rhee AC Olgac S Ohori M Russo P. Mucinous adenocarcinoma of the prostate: a case report of long-term disease-free survival and a review of the literature. Urology. (2004) 63:779–80. 10.1016/j.urology.2003.12.006 15072909

[8] Grignon DJ. Unusual subtypes of prostate cancer. Mod Pathol. (2004) 17:316–27. 10.1038/modpathol.3800052 14976541

[9] Samaratunga H Delahunt B Srigley JR Yaxley J Johannsen S Coughlin G. Mucinous adenocarcinoma of prostate and prostatic adenocarcinoma with mucinous components: a clinicopathological analysis of 143 cases. Histopathology. (2017) 71:641–7. 10.1111/his.13278 28590015

[10] Osunkoya AO Nielsen ME Epstein JI. Prognosis of mucinous adenocarcinoma of the prostate treated by radical prostatectomy: a study of 47 cases. Am J Surg Pathol. (2008) 32:468–72. 10.1097/PAS.0b013e3181589f72 18300802

[11] Zhao F Wang J Chen M Chen D Ye S Li X. Sites of synchronous distant metastases and prognosis in prostate cancer patients with bone metastases at initial diagnosis: a population-based study of 16,643 patients. Clin Transl Med. (2019) 8:30. 10.1186/s40169-019-0247-4 31784868 PMC6884608

[12] Salinas CA Tsodikov A Ishak-Howard M Cooney KA. Prostate cancer in young men: an important clinical entity. Nat Rev Urol. (2014) 11:317–23. 10.1038/nrurol.2014.91 24818853 PMC4191828

[13] Deng Y Bi R Zhu Z Li S Xu B Rather WA A surveillance, epidemiology and end results database analysis of the prognostic value of organ-specific metastases in patients with advanced prostatic adenocarcinoma. Oncol Lett. (2019) 8:1057–70. 10.3892/ol.2019.10461 PMC6607368 31423166

[14] Liu Y Xia Q Xia J Zhu H Jiang H Chen X. The impact of marriage on the overall survival of prostate cancer patients: a surveillance, epidemiology, and end results (SEER) analysis. Can Urol Assoc J. (2019). 13:E135–9. 10.5489/cuaj.5413 30332597 PMC6520053

[15] Epstein JI Amin MB Reuter VE Humphrey PA. Contemporary gleason grading of prostatic carcinoma: an update with discussion on practical issues to implement the 2014 international society of urological pathology (ISUP) consensus conference on gleason grading of prostatic carcinoma. Am J Surg Pathol. (2017) 41:e1–e7. 10.1097/PAS.0000000000000820 28177964

[16] Epstein JI Egevad L Amin MB Delahunt B Srigley JR Humphrey PA. The 2014 international society of urological pathology (ISUP) consensus conference on gleason grading of prostatic carcinoma: definition of grading patterns and proposal for a new grading system. Am J Surg Pathol. (2016) 40:244–52. 10.1097/PAS.0000000000000530 26492179

[17] Samaratunga H Delahunt B Yaxley J Srigley JR Egevad L. From gleason to international society of urological pathology (ISUP) grading of prostate cancer. Scand J Urol. (2016) 50:325–9. 10.1080/21681805.2016.1201858 27415753

[18] Manne RK Haddad FS. Mucinous adenocarcinoma of prostate. Urology. (1989) 33:247–9. 10.1016/0090-4295(89)90404-4 2537553

[19] Johnson H Zhou M Osunkoya AO. ERG expression in mucinous prostatic adenocarcinoma and prostatic adenocarcinoma with mucinous features: comparison with conventional prostatic adenocarcinoma. Hum Pathol. (2013) 44:2241–6. 10.1016/j.humpath.2013.05.006 23849895

[20] Steele CB Li J Huang B Weir HK. Prostate cancer survival in the United States by race and stage (2001-2009): findings from the CONCORD-2 study. Cancer. (2017) 123:5160–77. 10.1002/cncr.31026 29205313 PMC6077841

[21] Marcus DM Goodman M Jani AB Osunkoya AO Rossi PJ. A comprehensive review of incidence and survival in patients with rare histological variants of prostate cancer in the United States from 1973 to 2008. Prostate Cancer Prostatic Dis. (2012) 15:283–8. 10.1038/pcan.2012.4 22349984

[22] Guler OC Onal C Erbay G Bal N. Prostate mucinous carcinoma treated with definitive radiotherapy and hormonal therapy: case report and review of the literature. Clin Genitourin Cancer. (2014) 12:e43–6. 10.1016/j.clgc.2013.11.018 24365124

