# Supplementary material for: Mucinous Prostate Cancer Shows Similar Prognosis to Typical Prostate Acinar Carcinoma: A Large Population-Based and Propensity Score-Matched Study
Source: Front Oncol. 2020 Jan 9;9:1467. doi: 10.3389/fonc.2019.01467 (PMC6962295; doi:10.3389/fonc.2019.01467)
Supplement: Supplementary file 1 [file Table_1.DOCX]

sTable 1. The percentage of mucinous PCa relative to total PCa (n=360).

| **Year of diagnosis** | **Mucinous PCa** | **Total PCa** | **% mucinous PCa** |
| --- | --- | --- | --- |
| **2004** | 29 | 40790 | 0.07% |
| **2005** | 34 | 38713 | 0.09% |
| **2006** | 42 | 44923 | 0.09% |
| **2007** | 28 | 48428 | 0.06% |
| **2008** | 37 | 46443 | 0.08% |
| **2009** | 27 | 47677 | 0.06% |
| **2010** | 23 | 47382 | 0.05% |
| **2011** | 28 | 48616 | 0.06% |
| **2012** | 21 | 42089 | 0.05% |
| **2013** | 17 | 41430 | 0.04% |
| **2014** | 21 | 39350 | 0.05% |
| **2015** | 25 | 42330 | 0.06% |
| **2016** | 28 | 44785 | 0.06% |

Note: Mucinous PCa and total PCa: diagnosed by positive histology and labeled “one primary only”.

sTable 2. Univariate and multivariate analyses for OS of patients.

| **Variables** | **Level** | **Univariate** |  | **Multivariate** |  |
| --- | --- | --- | --- | --- | --- |
|  |  | **HR (95% CI)** | **P value** | **HR (95% CI)** | **P value** |
| **Age** | ≤65 | 1 |  | 1 |  |
|  | >65 | 4.551(2.483~8.340) | <0.001 | 4.258(2.270~7.988) | <0.001 |
| **Race** | White | 1 |  | / | / |
|  | Black | 0.988(0.443~2.201) | 0.976 | / | / |
|  | Others/Unknown | 0.293(0.040~2.128) | 0.225 | / | / |
| **Marital status** | Married | 1 |  | / | / |
|  | Unmarried | 1.626(0.867~3.050) | 0.130 | / | / |
|  | Unknown | 1.321(0.577~3.027) | 0.510 | / | / |
| **Tumor grade** | Low | 1 |  | / | / |
|  | High | 1.275(0.533~3.053) | 0.585 | / | / |
|  | Unknown | 12.142(4.538~32.488) | <0.001 | / | / |
| **PSA (ng/ml)** | <10 | 1 |  | 1 |  |
|  | ≥10~<20 | 0.669(0.229~1.958) | 0.463 | 0.830(0.280~2.462) | 0.738 |
|  | ≥20 | 3.171(1.600~6.285) | 0.001 | 1.349(0.549~3.318) | 0.514 |
|  | Unknown | 3.242(1.580~6.650) | 0.001 | 2.977(1.433~6.186) | 0.003 |
| **Summary stage** | Localized | 1 |  | 1 |  |
|  | Regional | 1.031(0.504~2.110) | 0.933 | 1.178(0.512~2.711) | 0.700 |
|  | Distant | 10.365(5.004~21.469) | <0.001 | 9.866(3.558~27.356) | <0.001 |
| **Gleason score** | ≤6 | 1 |  | / | / |
|  | 3+4 & 4+3 | 0.491(0.206~1.172) | 0.109 | / | / |
|  | ≥8 | 1.218(0.505~2.940) | 0.661 | / | / |
|  | Unknown | 1.008(0.408~2.492) | 0.986 | / | / |
| **Surgery** | Yes | 1 |  | 1 |  |
|  | None | 2.960(1.680~5.216) | <0.001 | 1.436(0.717~2.875) | 0.308 |
| **RT** | Yes | 1 |  | / | / |
|  | None/Unknown | 0.616(0.345~1.098) | 0.101 | / | / |

Note: OS: Overall survival; HR: Hazard ratio; Low tumor grade: Well and moderately differentiated tumor grade; High tumor grade: Poorly and undifferentiated tumor grade; PSA, prostate specific antigen; RT: Radiation therapy.
