# Supplementary material for: Mucinous Prostate Cancer Shows Similar Prognosis to Typical Prostate Acinar Carcinoma: A Large Population-Based and Propensity Score-Matched Study
Source: Front Oncol. 2020 Jan 9;9:1467. doi: 10.3389/fonc.2019.01467 (PMC6962295; doi:10.3389/fonc.2019.01467)
Supplement: Supplementary file 2 [file Table_2.DOCX]

**Figure legends in supplementary materials**

**sFig 1.** A: Kaplan-Meier estimated OS for patients with prostate adenocarcinoma and mucinous PCa (p=0.208); B: Kaplan-Meier estimated OS for localized, regional, and distant mucinous PCa (localized vs. regional: p=0.942; localized vs. distant: p<0.001; regional vs. distant: p<0.001); C: Kaplan-Meier estimated OS for mucinous PCa with and without surgery (p<0.001); D: Kaplan-Meier estimated OS for mucinous PCa with and without radiation (p=0.097).

**sFig 2.** Kaplan-Meier estimated CSS for mucinous PCa patients treated with surgery & RT after surgery, only surgery, only RT, and no surgery & RT (surgery & RT after surgery vs. only surgery: p=0.046; surgery & RT after surgery vs. only RT: p=0.241; surgery & RT after surgery vs. No surgery & RT: p=0.619; Only surgery vs. Only RT: p=0.592; Only surgery vs. No surgery & RT: p<0.001; Only RT vs. No surgery & RT: p=0.045).
