# Supplementary figures and images for: Mucinous Prostate Cancer Shows Similar Prognosis to Typical Prostate Acinar Carcinoma: A Large Population-Based and Propensity Score-Matched Study
Source: Front Oncol. 2020 Jan 9;9:1467. doi: 10.3389/fonc.2019.01467 (PMC6962295; doi:10.3389/fonc.2019.01467)

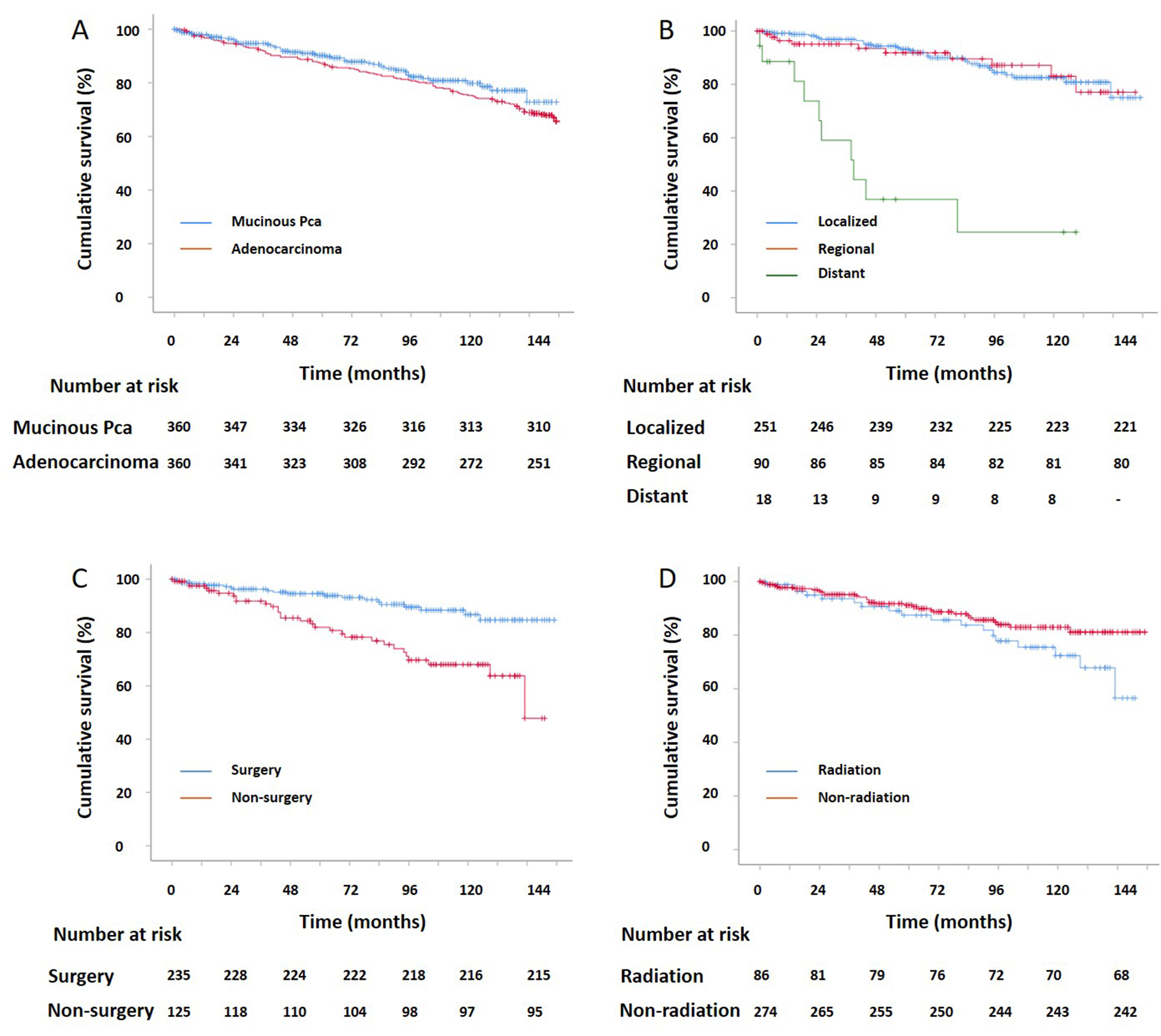

Supplement: Supplementary file 3 [file Image_1.JPEG]

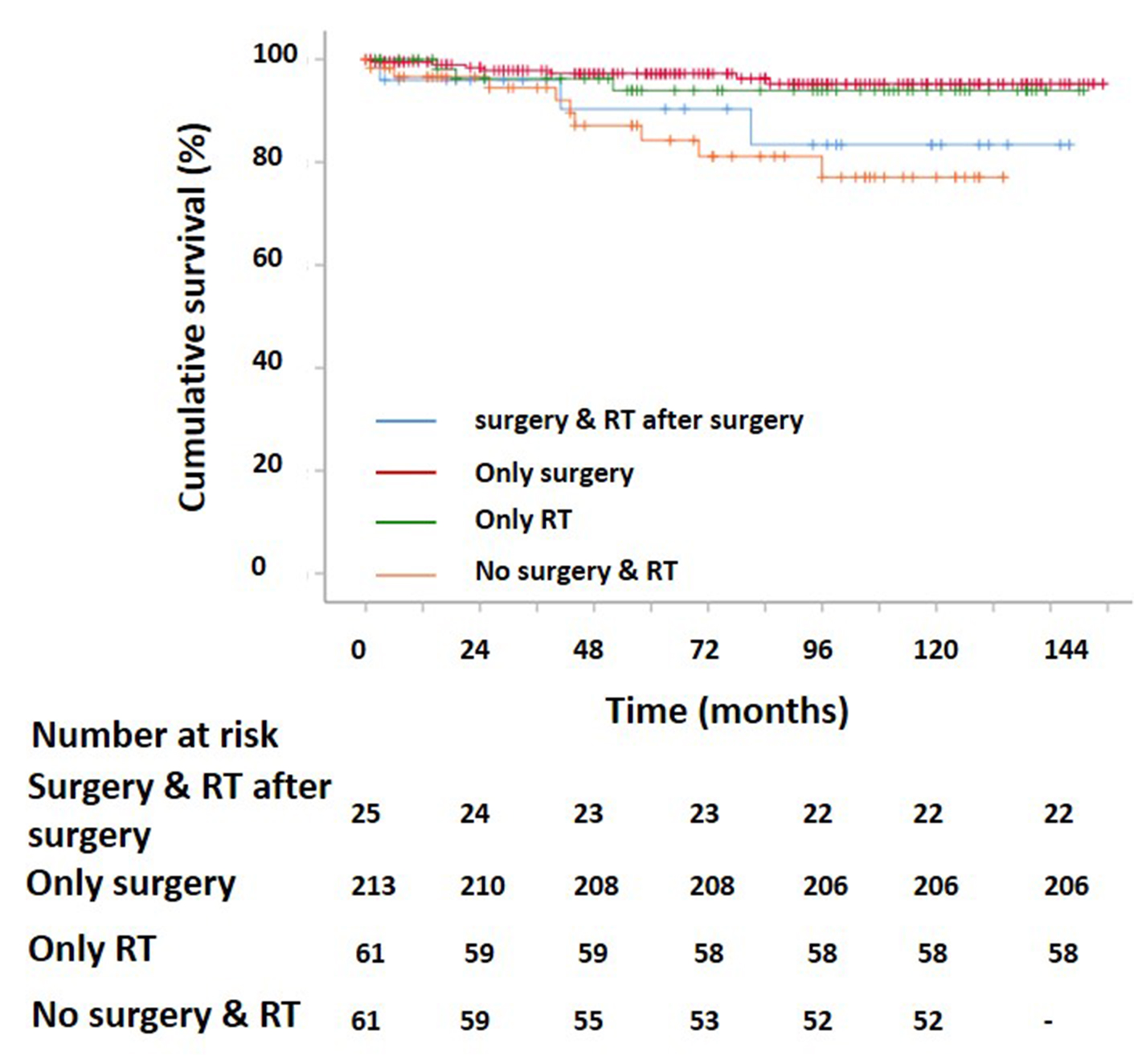

Supplement: Supplementary file 4 [file Image_2.JPEG]
